# Determining physiological responses of mussels (*Mytilus edulis*) to hypoxia by combining multiple sensor techniques

**DOI:** 10.1093/conphys/coaf023

**Published:** 2025-04-11

**Authors:** Emily Adria Peterson, Marinus Cornelis Keur, Michael Yeboah, Thomas van de Grootevheen, Luke Moth, Pauline Kamermans, Tinka Murk, Myron A Peck, Edwin Foekema

**Affiliations:** Department of Coastal Systems, Royal Netherlands Institute for Sea Research, Landsdiep 4, 1797 SZ, 't Horntje (Texel), The Netherlands; Marine Animal Ecology, Wageningen University & Research, Droevendaalsesteeg 1, Building 107, 6708 PB Wageningen, The Netherlands; Wageningen Marine Research, Ankerpark 27, 1781 AG, Den Helder, The Netherlands; Marine Animal Ecology, Wageningen University & Research, Droevendaalsesteeg 1, Building 107, 6708 PB Wageningen, The Netherlands; Marine Animal Ecology, Wageningen University & Research, Droevendaalsesteeg 1, Building 107, 6708 PB Wageningen, The Netherlands; Marine Animal Ecology, Wageningen University & Research, Droevendaalsesteeg 1, Building 107, 6708 PB Wageningen, The Netherlands; Wageningen Marine Research, Ankerpark 27, 1781 AG, Den Helder, The Netherlands; Marine Animal Ecology, Wageningen University & Research, Droevendaalsesteeg 1, Building 107, 6708 PB Wageningen, The Netherlands; Department of Coastal Systems, Royal Netherlands Institute for Sea Research, Landsdiep 4, 1797 SZ, 't Horntje (Texel), The Netherlands; Marine Animal Ecology, Wageningen University & Research, Droevendaalsesteeg 1, Building 107, 6708 PB Wageningen, The Netherlands; Marine Animal Ecology, Wageningen University & Research, Droevendaalsesteeg 1, Building 107, 6708 PB Wageningen, The Netherlands; Wageningen Marine Research, Ankerpark 27, 1781 AG, Den Helder, The Netherlands

**Keywords:** Biosensor, bivalve physiology; ecophysiology, coastal ecology

## Abstract

Intertidal bivalves survive longer without oxygen when aerially exposed during low tide than when submerged in hypoxic water. To understand this, we combined three biosensors to continuously monitor responses of individual blue mussels (*Mytilus edulis*) to aerial exposure in simulated low-tide conditions and during aqueous hypoxia. A valve sensor, heart rate monitor, and an in-shell oxygen microsensor simultaneously recorded behavioural and physiological responses. During aerial exposure, which often occurs in the intertidal, all individuals immediately closed their valves, rapidly depleted in-shell oxygen, and decreased their heart rate. This suggested a shift to anaerobic metabolism and reduced activity as mechanisms to save energy and survive in-shell anoxia during ‘low-tide’ conditions. At the onset of exposure to hypoxic (<1 mg O_2_/L) water, however, all mussels fully opened their valves, with 75% of the individuals increasing valve activity for at least 1 hour (the duration of our measurements), possibly in an attempt to collect more oxygen by increasing filtration activity. Only 25% of the mussels closed their valves after about 40 minutes of aqueous hypoxia, shifting to the energy efficient strategy used during aerial exposure. As the valves of most individuals remained open during hypoxia, a mussel does not appear to need to close its valve to begin the transition to anaerobic metabolism. Interindividual variation in responses was much lower after exposure to air compared to aqueous hypoxia when the heart rate of most mussels either steadily declined or became highly erratic. Differences in energy expenditure during these different types of exposures likely explains why most mussels, at least from the population we studied, can survive longer during exposure to air compared to aqueous hypoxia, a situation that could occur under situations of elevated temperature in waters with high nutrient loads.

## Introduction

Intertidal bivalves fulfil an integral functional and trophodynamic role in soft-sediment coastal environments, particularly as prey for both fish and birds foraging in intertidal habitats (Rullens *et al.,* 2019). Besides their value within the food web, bivalves provide numerous ecosystem services including provisioning services when harvested for food and regulating services such as nutrient remineralization, pollutant removal, and decreased turbidity through filter feeding (Pfister, 2007). Blue mussel (*Mytilis edulis)* is one of the most common bivalves within intertidal habitats around the world (Buschbaum *et al.,* 2009). In homogenous soft-sediment environments, intertidal mussels contribute to a more heterogeneous intertidal zone by providing stabilized habitats and altering biogeochemistry in the sediment (Widdows and Brinsley, 2002). As ‘ecosystem engineers’, mussels contribute to biodiversity by building habitats that provide shelter for benthic invertebrates and early life stages of fish (Buschbaum *et al.,* 2009; van der Zee *et al.,* 2012; Dame, 2016; Rullens *et al.,* 2019).

Decreases in the abundance of intertidal bivalves have been documented globally, with climate change and other anthropogenic activities understood as driving forces for their decline (Gillies *et al.,* 2018; Seuront *et al.,* 2019; Salmond and Wing, 2023). Numerous changes have occurred in intertidal ecosystems, including altered salinity, increased temperature and increased nutrient loads leading to decreased oxygen levels and ultimately hypoxia (Domínguez *et al.,* 2023; Seibel, 2023). Warming and hypoxia often occur together especially under eutrophic conditions and represent a synergistic stress for intertidal bivalves (Cheng *et al.,* 2015; Fey *et al.,* 2015; Bible *et al.,* 2017; Salmond and Wing, 2023). Heatwaves can be particularly destructive to shallow water communities (Garrabou *et al.,* 2022), and if the bivalves are already threatened due to other stressors, their ecosystem services will also be threatened.

Intertidal mussels, like other bivalves, have both aerobic and anaerobic metabolisms, which allow individuals to survive long periods exposed to air during low tide. Upon aerial exposure, mussels close their valves, and oxygen concentrations in the body cavity quickly decrease, with anaerobic metabolism commencing at the onset of hypoxia (Livingstone and Bayne, 1977). Anaerobic respiration utilizes glucose and aspartate to produce succinate and alanine, referred to as the glucose-succinate and aspartate-succinate pathways, respectively (Isani *et al.,* 1995). Within minutes of being re-submerged, mussel valves reopen and heart rate increases as normal aerobic physiological mechanisms restart (Ellington, 1983). While intertidal mussels experience 50% mortality after 70 hours of aerial exposure, aqueous hypoxia causes 50% mortality within 50 hours (Altieri, 2006). This suggests mussels follow a different strategy during aerial exposure in low-tide conditions than during aqueous hypoxia (Altieri, 2006).

Biosensors have become prevalent in physiological studies, with oxygen biosensors common for measuring both ambient oxygen in environmental conditions and within animal tissues (Bagshaw *et al.,* 2011; Russnak *et al.,* 2021; Talevi *et al.,* 2023). While numerous types of electrochemical oxygen sensors have been developed, fibreoptic microsensors are most commonly used for ecophysiology (Bongaerts *et al.,* 2011; Tagliarolo and McQuaid, 2015; Barott *et al.,* 2020). While heart rate monitors have been used on bivalves previously (Bakhmet *et al.,* 2019; Xing *et al.,* 2019; Bakhmet *et al.,* 2021), recent advances have created heart rate monitors engineered for easier application and higher quality measurements in bivalves. Valve monitoring for bivalves has remained steadfast since the 1990s, with the so-called ‘Mosselmonitor’ (mussel monitor) used most often (Curtis *et al.,* 2000; Kramer and Foekema, 2001; Barile *et al.,* 2016). Throughout years of development, the reduction of biosensor size now allows multiple sensors to be simultaneously applied to small animals such as bivalves.

The aim of this study was to compare the behavioural and physiological responses of mussels to aerial exposure in simulated low-tide conditions, and during aqueous hypoxia. For this, we simultaneously monitored valve movement, in-shell oxygen levels, and heart rate of individual mussels. In previous experiments, only single biosensors, often referred to as ‘microsensors’, have been deployed in mussels, except for two studies where oxygen and valve sensors were simultaneously deployed (Curtis *et al.,* 2000; Tang and Riisgård, 2016). As far as we are aware, our study is the first to use three biosensors on individual bivalves to obtain simultaneous behavioural and physiological data.

## Materials and Methods

### Sampling and animal maintenance

Blue mussels *Mytilus edulis* were collected from a wild colony within the intertidal area ‘*The Balgzand’* in the southwestern part of the Wadden Sea (Supplementary Fig. S1). Mussels were collected in May 2023 for aerial exposure experiments and in November 2023 for aqueous hypoxia experiments. The wild colony was attached to (artificial) rocky substrate in an intertidal zone close to shore. Healthy mussels, characterized by the absence of visible shell injuries, were selected for the experiments. After collection, mussels were transported within 10 minutes in an isolated bucket to the laboratory (Wageningen Marine Research in Den Helder, the Netherlands). Mussels were subsequently cleaned from epibiotic shell growth and separated into two 15-L aquaria with 10 bivalves each to allow them to adapt to test conditions for 3 days prior to the experiment. The aquaria were filled with natural seawater, kept at a constant temperature of 18°C, >99% air saturation (9.6 mg/L), 33 parts per thousand salinity, and 7.80 pH with mussels totally submerged before transfer to the experimental setup. Mussels were fed twice daily, monocultures of *Isochrysis galbana* algae cultures were added to the aquaria twice daily to maintain a concentration of 100 000 cells/ml. The mussels chosen for each experiment were approximately the same size and weight (Table 1). All experiments carried out in this research followed legislation and guidelines for animal experimentation in the Netherlands. However, as invertebrate experimentation is not regulated, we did not require any permits or species approvals.

**Table 1:** Average length (measured along the longest anteroposterior axis) and fresh weight ± standard deviation (range) for each sample collection

**Experiment**	**Collection**	**Length** (mm)	**Fresh weight** (g)
1a, 1b (aerial exposure)	May 2023	53 ± 2 (51–56)	17.4 ± 5.0 (9.9–21.8)
2, 3 (aqueous hypoxia)	November 2023	52 ± 4 (47–60)	16.6 ± 4.0 (11.7–23.9)

### Biosensors

#### Oxygen sensor

The PreSens Profiling Oxygen Microsensor PM-PSt7 (PreSens, 2024) was used to measure in-shell oxygen levels. The night prior to measurements, a 0.5-mm hole was carefully drilled into the shell of each mussel to allow for the microsensor to be placed near the mantle for optimal oxygen measurements in the shell. In earlier pretrials, this small hole caused no behavioural changes nor physiological alterations in the bivalves. The oxygen sensors were calibrated before and after measurements to log any discrepancies between individual microsensors. The microsensor took one reading every 10 seconds, with readings recorded in the PreSens software package (PreSens Measurement Studio 2, version 3.0.3.1703).

#### Valve sensor

The laboratory version of the ‘Mosselmonitor’ (Aquadect, Nieuwerkerk, The Netherlands; (Aquadect, 2024)) was used to monitor the valve activity in each mussel. The sensor consists of two probes that measure the distance between each shell via a kHz signal that is sent from the front to the back valve sensor. This results in a voltage reading that the Mosselmonitor software (PresentIT 3 version 3.0.1.12) translates proportionally to the distance between the front and back valve sensors. Mosselmonitor raw voltage readings are in a linear relationship with true valve distance; therefore, valve distance values were scaled between 0—the value of shell closure and 1—the maximum valve opening position shown during experimental trials (Kramer and Foekema, 2001). The Mosselmonitor took one reading every 10 seconds, aligned with the PreSens oxygen sensor measurements. The Mosselmonitor sensors were first covered with waterproof adhesive bandage tape (Leukoplast) and then glued onto cleaned and dried shells.

#### Heart rate sensor

The ElectricBlue ‘Pulse V2’ heart rate monitor was used to measure the heart rate of each mussel. The sensor is a heart frequency logger specifically manufactured to measure cardiac performance of molluscs and crustaceans (ElectricBlue, 2024). The sensor consists of an infrared (IR) sensor that needs to be glued to the shell right above the heart, requiring the user to utilize precision in application (Fig. 3). The same glue and plaster were used for both the valve and heart rate sensors. The IR sensor logs 10 readings per second, from which peaks in amplitude can be counted to calculate heart rate.

**Figure 1:**
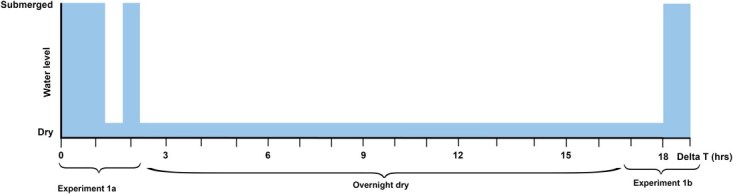
Graphic representation of experiment 1a and 1b

**Table 2:** Overview of aerial exposure experimental protocol

	**Minutes in experiment**	**Mussels position**	**Disturbance**	**Monitoring**
Experiment 1a				
Rest period	0–30	Submerged	None	Valve, HR, O_2_
	30–35	Submerged	Tapping	Valve, HR, O_2_
	35–75	Submerged	None	Valve, HR, O_2_
	75–115	Emerged	Exposure to air	Valve, HR, O_2_
Recovery period	115–145	Submerged	None	Valve, HR, O_2_
				
**Overnight dry**	17 hours	Emerged	Exposure to air	N/A

Experiment 1b				
Start recording	0–60	Emerged	Exposure to air	Valve, HR
Recovery period	60–120	Submerged	None	Valve, HR

### Experimental designs

#### Aerial exposure experimental protocol

The aerial exposure experiment was performed with five mussels positioned in individual full glass 10-L aquaria (25 × 25 × 20 cm). To ensure that the mussels were fully exposed to air when the water was syphoned out of the aquaria, mussels were placed about 3 cm above the aquarium floor on a polyethylene wire construction. Before being transferred to the aquaria, each mussel was equipped with valve sensors, an in-shell oxygen sensor, and a heart rate sensor. After the mussels were in position, the aquaria were filled with 0.45-μm-filtered aerated natural North Sea water enriched with a concentration of approximately 100 000 cells/ml of cultivated monoculture of microalgae (*Isochrysis galbana*) to stimulate the mussel’s filtration activity. Gentle aeration was added on one side of the aquarium, ensuring that the bubble stream did not reach the mussel positioned at the opposite side.

After approximately 1 hour of acclimatization, experiment 1a commenced with valve position, heart rate and in-shell oxygen concentrations were monitored for 30 minutes without disturbance (Fig. 1; Table 2). Next, each aquarium was tapped with metal tweezers in an attempt to stimulate a stress reaction and ensure responsiveness, causing mussels to close their valves. Afterwards, the mussels were left undisturbed for 40 minutes. The water from each aquarium was then emptied using a syphon to fully exposure the mussels to air for 40 minutes. The aquaria were filled with seawater, and the mussels were left undisturbed for another 30 minutes. After this 30-minute recovery period, the aquaria were drained, oxygen sensors were removed, and the mussels were returned to the aquarium on the wire above the water surface. Oxygen sensors were removed as they are rather fragile and might not withstand an overnight period inside the shell of the mussel without altering the validity of the sensor. The oxygen sensors were not reinserted to avoid introducing a stress bias to the mussels. The mussels remained exposed to air overnight, and monitoring of valve position and heart rate was started again the next morning (indicating start of experiment 1b). After monitoring for 1 hour, the aquaria were filled with seawater and monitoring continued for another 2 hours (Fig. 1).

### Aqueous hypoxia experimental protocol

**Figure 2:**
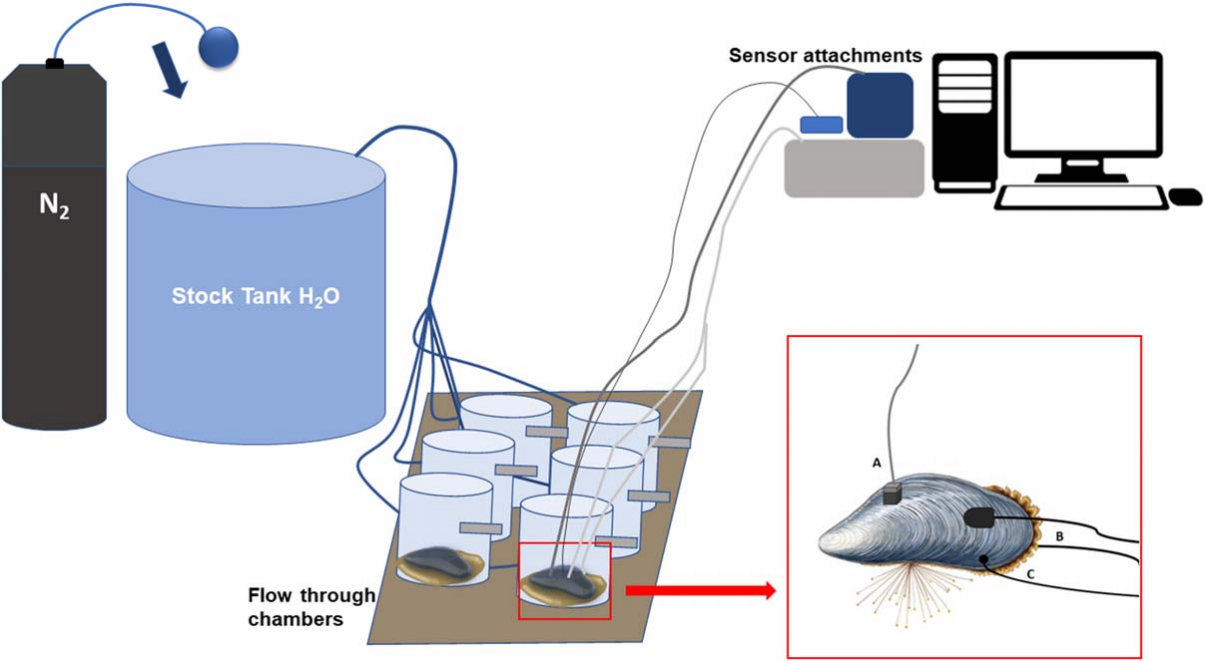
Graphic representation of setup for the aqueous hypoxia experiment in flow-through chambers with *M. edulis* (**A**: heart rate sensor, **B**: front and back valve sensor, **C**: oxygen microsensor)

The experimental setup for the aqueous hypoxia experiment featured one stock tank (approximately 80 L) and six flow-through chambers (volume 180 ml, diameter 7 cm) (Fig. 2). The seawater entered the flow chamber near the bottom and exits on the other side at the top of the chamber. The stock tank was filled with natural seawater and was aerated for 12 hours before the experiment and kept at 18°C, 33 parts per thousand salinity, and 7.90 pH. Twelve hours before treatment, valve and heart sensors were glued onto the mussels selected for the experiment. The in-shell oxygen sensor was applied 2 hours before the experiment. One individual mussel was placed in each of the six flow-through chambers (Fig. 2). Three hours before, the start of the experiment water in the stock tank was enriched with a concentration of approximately 100 000 cells/ml of cultivated monoculture microalgae (*Isochrysis galbana*) to stimulate the mussel’s filtration activity. Mussels equipped with all three biosensors were placed into the flow through chambers and allowed to acclimatize for 1 hour before the experiment began.

Monitoring the physiology of mussels exposed to aqueous hypoxia was conducted in two experiments. All experiments started with a baseline measurement of all three parameters for mussels maintained in flow-through, oxygen-saturated water conditions. Hypoxic conditions were simulated after 75 minutes (Table 3) by stopping the aeration of the stock tank and bubbling nitrogen gas into the stock tank. Oxygen levels in the stock tank were measured every minute with a Hach HQ4200 oxygen probe until the tank reached stable <1 mg O_2_/L within 10 minutes, after which oxygen measurements were reduced to 5-minute intervals. The hypoxic conditions were maintained for 1 hour and 45 minutes. The second experiment began the next day with a new set of six mussels and a hypoxic period of 75 minutes. During the first experiment, no heart rate data were collected due to a technical failure. During the second experiment, data were collected with all three sensors.

**Table 3:** Overview of protocol for aqueous hypoxia experiment

	**Minutes in experiment**	**Mussels position**	**Disturbance**	**Monitoring**
Experiment 2				
Rest period	0–75	Submerged	None	Valve, O_2_
	75–180	Submerged	Hypoxic water	Valve, O_2_
Experiment 3				
Rest period	0–75	Submerged	None	Valve, HR, O_2_
	75–150	Submerged	Hypoxic water	Valve, HR, O_2_

### Data processing

Raw data from each sensor were processed before combining them for visualizations and statistical analyses. Raw timestamps were converted to elapsed time expressed in minutes to align readings from all three sensors. Oxygen sensor readings were standardized using a calibration factor calculated from the pre- and postcalibration tests. As the Mosselmonitor raw values are expressed in millivolts, values were normalized from 0 to 1 based on the min and max value measured for each individual. Thus, on a normalized scale, 0 = fully closed valve and 1 = fully opened valve.

Raw amplitude values from the heart rate monitor were processed in RStudio v4.3.1 to count the cyclical peaks of the amplitude to calculate heart rate. Heart rate was calculated from raw amplitude data using a custom algorithm that uses the signal, oce and dplyerpackages in R. Heart beats were automatically counted using a zero-crossing counting algorithm. Zero crossing methods are robust for counting rhythmic beats such as heart rates against background noise (Kohler *et al.,* 2003; Khan *et al.,* 2013). To determine zero crossing, out-of-range values were removed and a moving average (time window equal to 50 seconds) was used to estimate the mean, centering the signal along the horizontal axis (amplitude = 0). Each subsequent amplitude value was subtracted from the local mean established from the moving 50-second window to create the zero-crossing line. To count as a major peak to be included in beats per minute, a beat must have a zero crossing, a maximum peak, another zero crossing and a final minimum value to be included. Fast Fourier Transform, a noise reduction high-pass filter, was utilized to filter out very high-frequency (>3.5 Hz) components of the signal. With this cleaning or preprocessing of the signal, the zero crossings could then be identified, and the amplitudes and wave periods were derived. This method was thus used to count major peaks, ignoring the minor fluctuations present from the heart rate monitor. Utilizing a 50-second moving window to calculate bpm results in numerous bpm estimates per minute, as the heart rate monitor takes 10 readings per second. The final bpm value extracted per minute is the minimum of the overlapping bpm estimates. This approach avoids outlier bpm estimates as the sensor attached from the bivalve can experience spikes of activity from the IR sensor as the bivalve moves in the flow-through chamber (mostly from valve movement during feeding/respiration). These spikes can create false beats and background noise, so the minimum BPM estimate created the most accurate final BPM value for all experimental setups used in this study. Raw visuals from the custom algorithm showing how beats are counted in the zero-crossing algorithm can be found in Supplementary Fig. S2. Furthermore, a random manual accuracy assessment for each mussel per experiment was conducted to ensure proper counting of peaks.

### Statistical analysis

For all experiments, the approach for data analysis was to compare the data collected during the prestressor phase (submerged and oxygenated) to the data collected during the respective stress of aerial exposure or aqueous hypoxia for each individual. The Shapiro–Wilk test was conducted to determine if the data from the heart and valve biosensor were normally distributed. As all datasets were determined to be non-normal, differences in heart rate (measured in beats per minute) and valve activity were analysed using the nonparametric Kruskal–Wallis test (Ding *et al.,* 2021; Pastorino *et al.,* 2021). The Dunn’s post hoc test was used to identify which time periods were statistically significant in comparison with the nonstress period (oxygenated submerged conditions), with *P* value adjusted by the Bonferroni correction (Ding *et al.,* 2022).

Using Kruskal–Wallis and Dunn tests, statistical significance of bpm and valve activity per individual were compared. Heart rate during the nonstress period was compared with the disturbance period (hypoxia, low-tide emergence) of each experiment in 10-minute intervals for heart rate and 5-minute readings for valve position. The difference in intervals for statistical analysis was due to the valve sensor taking one reading per 10 seconds, whereas heart rate was one reading per minute, necessitating a longer interval for analysis to allow for proper sample size. Fluctuations of heart and valve activity were compared to identify the temporal durations of each stressor that significantly impacted the valve and heart activity. Statistical significance was set at *P* value <0.05. All analyses were performed using RStudio version 4.3.1. using packages dplyr, readxl, stringr, openxlsx, and dunn test. Graphpad Prism v.10.1.2 was used to create visualizations to compare physiological activity between individuals.

### Results

#### Aerial exposure

**Figure 3:**
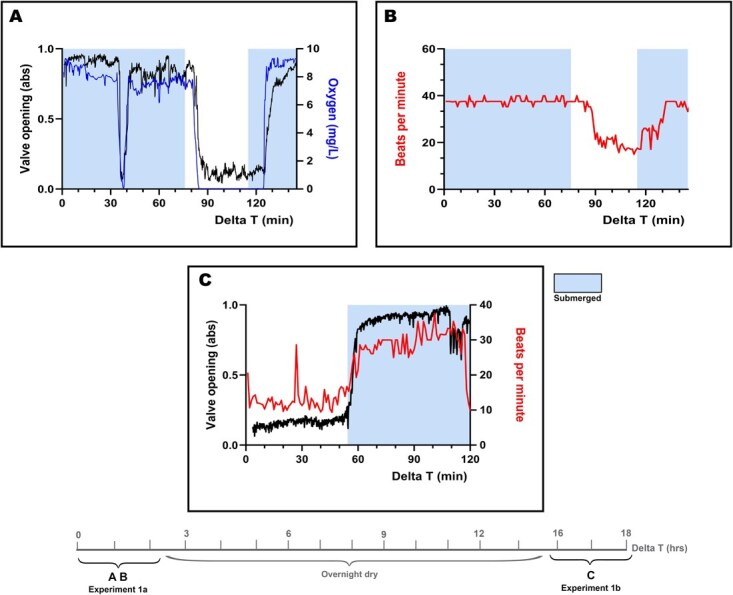
Responses of five *M. edulis* to median short-term and long-term aerial exposure (**A**: valve movement and in-shell oxygen level (mg/L) during short-term aerial exposure, **B**: heart rate during short-term aerial exposure, **C**: valve movement and heart rate during long-term aerial exposure). Presented data are averages from five mussels. The timeline underneath the figures to the moment of the data collection in reference to Fig. 1. For full per mussel breakdown, see Supplementary Material Figs S3 and S5

Results from the aerial exposure experiments revealed two baseline physiological modes when a mussel is submerged or aerially exposed. Since mussels showed a uniform response to aerial exposure across all individuals, mean values are presented in Fig. 3 (for individual data see Supplementary Figs S3 and S5). When submerged in oxygenated water, valve movements were relatively active with positions spanning 75% of the maximum opening during most of the period. When mussels were submerged, in-shell oxygen levels reflected the surrounding water conditions. While submerged during the tapping disturbance, all mussels closed valves immediately to the perceived disturbance; however, no heart response was detected (Fig. 3—delta T 38 minute). In-shell oxygen levels dropped to 0 mg/L oxygen within 2 minutes in response to the valve closure.

As soon as the water was removed and mussels were aerially exposed in experiment 1a, all mussels closed their valves within 10 minutes. Valve activity changes were statistically significant for all mussels during periods of aerial exposure due to this distinct valve closure (for all mussels, *P <* 0.0132; see Supplementary Fig. S4 for individual significance for valve closure during experiment 1a). After mussels closed their valves, in-shell oxygen concentrations were depleted within a few minutes. When oxygenated water was provided to the tanks, all mussels opened their shells and in-shell oxygen levels represented the oxygenated water conditions within 10 minutes. These responses were similar for all mussels after short (40 minutes in experiment 1a), and long (17 hours in experiment 1b) aerial exposure (Fig. 3). One mussel (mussel B, Supplementary Fig. S5) was unable to be included in the statistical test for valve activity in experiment 1b due to a malfunctioning of the sensor during the first 55 minutes.

**Figure 4:**
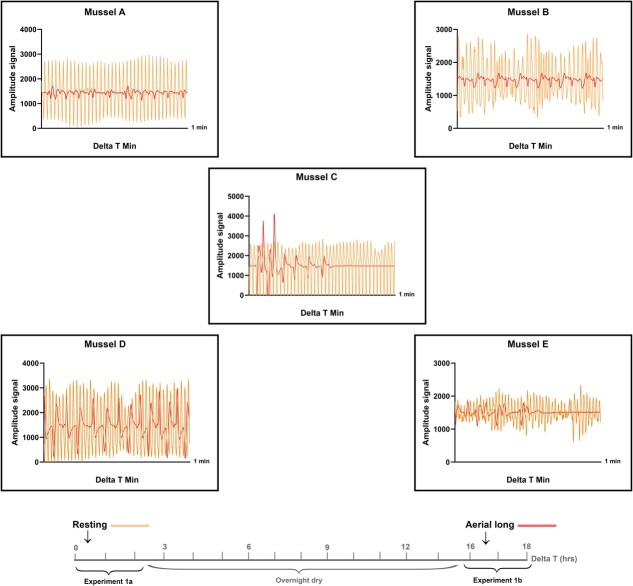
Range of amplitude (signal strength) during experiment 1a and 1b for all mussels

During submerged oxygenated conditions at the start of experiment 1a, the mean heart rate was 36 bpm (Table 4). There was a significant decrease in heart rate for all mussels during both short- and long-term aerial exposure (for all mussels, *P <* 0.0436; see Supplementary Figs S3 and S5 for individual significance). A rapid drop in heart rate was recorded after the valve closure at the onset of aerial exposure. Compared to when submerged, heart rate decreased, on average, by 40% during the first 30 minutes of aerial exposure, to a mean value 21 bpm. After long-term (overnight) exposure to air, heart rate decreased by an average of 62%, to a mean value heart rate of 13 bpm (Table 4). When resubmerged in fully oxygenated water, the mean heart rate increased to 29 bpm. Raw amplitude levels from the sensor signal changed cyclically with each experimental phase for aerial exposure, as depicted in Fig. 4.

**Table 4:** Heart rate (beats per minute) during experiments 1 and 3

**Environment**	**Average heart rate**	**Average percent change from prestress conditions**
Experiment 1 (aerial exposure)		
Submerged oxygenated—exp 1a	36 ± 4	n/a
Short-term aerial exposure—exp 1a	21 ± 5	**−40%**
Long-term aerial exposure—exp 1b	13 ± 2	**−62%**
Recovery—exp 1b	29 ± 8	**−18%**
Experiment 3 (hypoxia)		
Submerged oxygenated—exp 3	27 ± 5	n/a
Aqueous hypoxia—exp 3	19 ± 2	**−31%**

In summary, when mussels were submerged in oxygenated water, the physiological response was characterized by open and active valves, elevated and regular heart rate, and oxygen saturated tissues. When aerially exposed, valves immediately closed, in-shell oxygen rapidly decreases to 0% within 2 minutes, and heart rate is reduced (Fig. 3). Overall, all mussels showed only slight individual difference, with physiological modes during prestressor submerged and aerial exposure fairly uniform across individuals. It should be noted that the baseline heart rate during oxygenated submerged conditions in aqueous hypoxia experiments performed in November was 27 bpm as opposed to an average of 36 bpm in the aerial exposure experiment performed in May, while both experiments were performed at the same temperature (Table 4). This difference probably illustrates seasonality, as heart rate is lower during autumn and winter seasons than in spring and summer (Hagger *et al.,* 2010; Bakhmet *et al.,* 2019; Han *et al.,* 2020; Li *et al.,* 2021).

### Aqueous hypoxia

**Figure 5:**
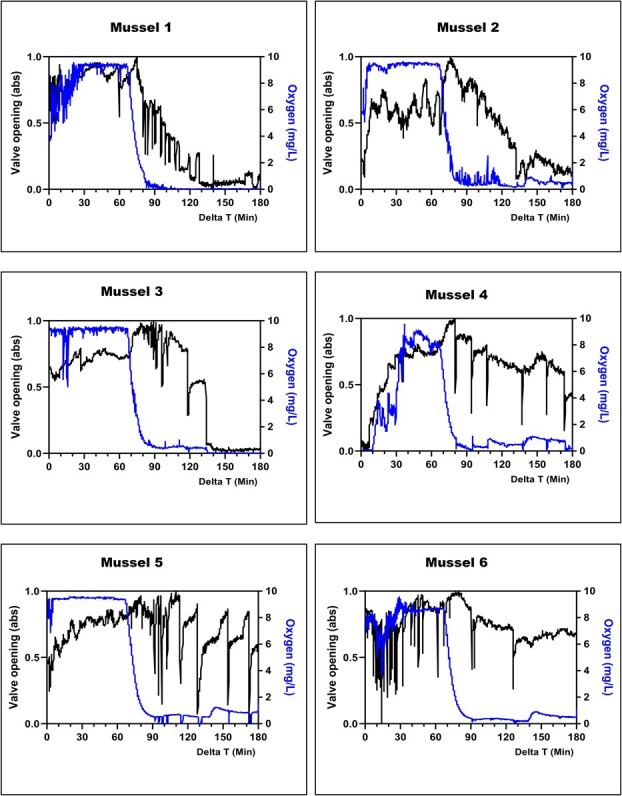
Mussel valve activity and in-shell oxygen concentration during submerged oxygenated (0–75 min) and aqueous hypoxic conditions (75–180 min)—experiment 2

**Figure 6:**
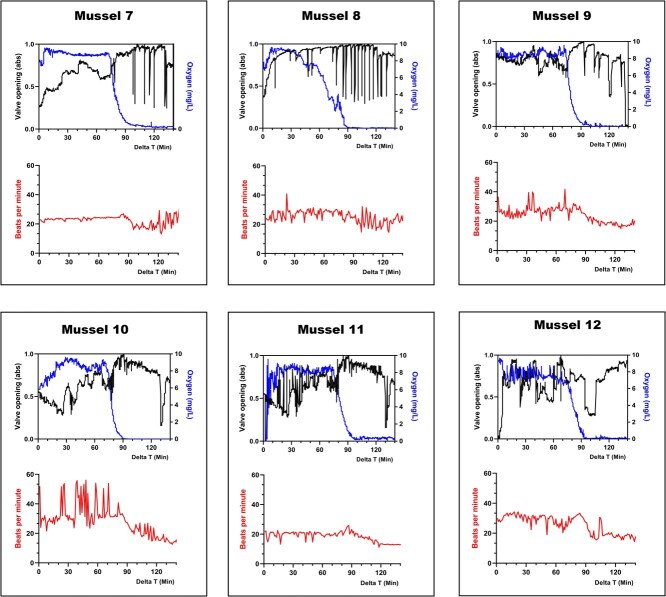
Mussel valve activity, in-shell oxygen concentration, and heartbeat per minute during submerged oxygenated (0–70 min) and aqueous hypoxic conditions (70–150 min)—experiment 3

Interindividual variability in physiological response was higher during aqueous hypoxia than aerial exposure. However, general responses were observed across all mussels for valve activity, in-shell oxygen, and heart rate. The most common response was a fast opening of the valve to the fullest extent at the onset of oxygen decrease. For 11 out of 12 mussels, this opening was followed by an increase in the frequency of valve movements throughout the hypoxic period (Figs 5 and 6). Valve activity during the first 10 minutes of hypoxia was statistically different than that observed during the oxygenated period for both experiments 2 and 3 (for all mussels, *P <* 0.0113; see Supplementary Figs S7 and S8 for individual significance).

In the first hypoxia experiment, after 55 minutes of hypoxia (130-minute Delta T in Fig. 5) when oxygen levels had dropped below 1 mg/L, three mussels markedly reduced valve activity and almost completely closed their valves (Fig. 5; mussels 1, 2 and 3). This behaviour was not observed in any mussels in the second hypoxia experiment. (Fig. 6). Besides these three mussels, the remaining mussels in both hypoxia experiments continued to have active valve movement throughout the hypoxic period. This response, in combination with no mortality occurring after exposure to 100 minutes of confirmed aqueous hypoxia, suggested a switch to anaerobic respiration. This further suggests that a mussel does not need to close its valve to begin the transition to anaerobic metabolism. For the mussels that did not close their valves during the oxygen depletion, in-shell oxygen sensors reflected the oxygen conditions of the surrounding water as the hypoxic water continued to flush over the mantle where the oxygen microsensor had been placed.

Unlike during aerial exposure, heart rate patterning during aqueous hypoxia was different for every individual. Heart rate decreased on average by 31% from 27 to 19 bpm during hypoxic water conditions. Heart rates were significantly altered for all mussels in comparison with the oxygenated period after 40 minutes of hypoxic conditions (Delta *T* = 115 in Fig. 6), when oxygen levels had dropped below 1 mg/L (for all mussels, *P <* 0.0484*;* see Supplementary Fig. S8 for individual significance). However, while this significant difference was observed for all mussels, significant changes differed per individual mussel. For example, mussel 7 increased heart rate fluctuations after 40 minutes of hypoxic water conditions, while mussel 10 decreased heart rate (Fig. 6).

## Discussion

This study demonstrated that, when aerially exposed, *M. edulis* exhibited a uniform response, characterized by immediate valve closure, oxygen depletion, and reduced heart rate. In contrast, during aqueous hypoxia, active valve movement continued throughout the hypoxic period, and heart rate patterning differed among individuals.

The general and well-known response of mussels to aerial exposure is immediate shell closure. This response is typical of intertidal shellfish as a strategy to avoid predation and desiccation (Widdows and Shick, 1985; Shick *et al.,* 1986). When closing its shell, the mussel isolates its tissue from the environment. Any oxygen in the water trapped inside the shell is completely depleted within minutes (as we observed here). This oxygen depletion mandates a switch to the ‘relatively energy expensive’ anaerobic metabolism, which results in a reduced heat rate (Babarro and De Zwaan, 2008). Our tri-sensor approach confirmed this response, where valve closure was immediate upon aerial exposure, in addition to an immediate and total depletion of in-shell oxygen levels to near 0 mg/L. Heart rate was reduced by 40% after mere minutes of aerial exposure and 62% after hours of aerial exposure.

The general response of the mussels to aqueous hypoxia was to open the shell to its maximum range at the onset of oxygen depletion, followed by a spike in valve activity. This behaviour may be related to an increase of the filtration rate (Riisgård *et al.,* 2003; Wilson *et al.,* 2005; Ahmmed *et al.,* 2021) that, in this situation, constitutes a useless attempt of the mussel to increase oxygen availability. Such an increase in respiration has been observed in other intertidal species experiencing low-oxygen conditions (Stevens and Gobler, 2018). In other situations, increased valve activity can be attributed to factors such as particle concentration, turbidity, diet type or salinity (Riisg**å**rd and Randløv, 1981; Newell *et al.,* 2001; Bucci *et al.,* 2008). However, as the experimental design had stable conditions per flow-through chamber for the aforementioned factors, the only plausible explanation is an attempt to increased filtration activity to maximize oxygen uptake. Furthermore, the behavioural and physiological response to oxygen depletion was more gradual and not as stark or rapid as the switch observed during aerial exposure. This observation aligns with the theory that mussels are able to smoothly transition from aerobic to anaerobic metabolisms as opposed to an abrupt ‘on/off’ switch (Livingstone and Bayne, 1977). The anaerobic metabolism of *M. edulis* has been estimated to be about 18 times less efficient than the aerobic metabolism (Gosling, 2008), which, therefore, cannot be maintained for a long period of time, especially in combination with the increased activity we observed for most of the mussels. Theoretically, mussels should be able to survive aqueous hypoxia much longer if they employed the strategy used during aerial exposure—to close valves and reduce heart rate. However, the responses we observed appear to be a suboptimal strategy to survive hypoxia. Biochemical analysis will need to be included in future studies to understand the energetic consequences associated with the behaviour observed in our experiments. These results align with the longer survival times during aerial exposure as opposed to aqueous hypoxia (Altieri, 2006). The results of the experiment demonstrate that low or negligible oxygen levels may not be a trigger for mussels to close valves.

While observed heart rates were similar across most individuals during aerial exposure, individual variation was particularly high for one mussel whose heart rate response was erratic and higher than the other mussels (Supplementary Fig. S3—mussel E). Another mussel had only relatively small and nonsignificant changes in valve activity in response to short-term aerial exposure during experiment 1a (Supplementary Fig. S3—mussel A). Individual variation in cardiac response to 40 minutes of aqueous hypoxic conditions could be categorized in two groups, those that decreased heart rate and those that increased heart rate fluctuations. The heart rate of the latter group oscillated between 15 and 30 bpm. These fluctuations align with ‘burst activity’ interspersing bradycardia during aqueous hypoxia in the subtropical mussel *Perna viridis* (Nicholson, 2002). No relationship was found between the fresh weight and length of the mussels in relation to individual differences. These individual differences may result from the adaptive strategies employed to contend with chronic or acute levels of stress, events occurring over an individual’s lifespan impacting both the stress response and overall health of the individual, as well as genotypic differences (Torossian *et al.,* 2020; Li *et al.,* 2021). However, across all experiments, long-term aerial exposure resulted in the most drastic reductions in heart rate, from 36 to 13 bpm after overnight (17 hours) of aerial exposure. However, average heart rates during aqueous hypoxia (19 bpm) remained lower than short-term aerial exposure (21 bpm) despite open and active valves throughout the hypoxic period.

We combined three biosensors to monitor individual physiological responses in the mussel *M. edulis* on a fine temporal scale. This approach represents a step forward in bivalve ecophysiology to recentre research around the individual as opposed to the group. Individual investigations are paramount to avoid oversight when seeking to understand the range of adaptive capacities present within a species. For example, aerial exposure represented a stressor where physiological variation for valve and heart activity was clear-cut across most individuals, reducing the importance of individual difference for that particular stressor. However, aqueous hypoxia represented a stressor that had a clear trend for valve activity, yet heart activity had numerous individual differences, which might have consequences for the ability to cope with this stressful condition in the long term. The mussels used for these experiments were collected from the field during low tide when they were emerged, as such it is evident that all mussels were familiar with periods of aerial exposure. Some type of adaptation, or perhaps even selection, could explain the rather uniform response to aerial exposure we observed. Aqueous hypoxia, on the other hand, is a condition that the mussels from this colony probably had never experienced, which might explain the variety of responses to this specific stressor. As such, we cannot assume that a stressor will elicit the same response from all individuals within a species or within the same population. We recommend that biosensors be used to reveal potential interindividual differences in the response to environmental stressors. Scaling up our study to include a larger number of individuals will help estimate the true range of responses present within a species and the magnitude of interindividual and/or seasonal variation in physiology. In addition, future studies would do well to include additional factors often present during hypoxia, such as increased pCO_2_ levels.

Shellfish restoration is already commonplace in European coastal areas (Pogoda *et al.,* 2019; Nielsen *et al.,* 2021; Hughes *et al.,* 2023) and this practice will likely increase in response to the European Nature Restoration Law passed in June 2024, which mandates at least 20% of Europe’s marine and terrestrial areas be actively restored by 2050. Within this law, restoration must be ‘climate smart’, acknowledging interacting local stressors to ensure sustainable restoration (Gissi *et al.,* 2021). Only when choosing habitat-forming species that can tolerate projected climate-driven changes in the environment can one effectively use restoration to support climate change mitigation and adaptation (Bulleri *et al.,* 2018). Considering that all efforts of mussel restoration in the Wadden Sea has been deemed nonresilient (de Paoli *et al.,* 2015), novel information is needed to understand the adaptive capacity of mussels to future environmental changes such as daily aerial emersion in warming temperatures of intertidal beds or more frequent exposure to aqueous hypoxia in subtidal beds under warmer and more eutrophic conditions (O’Neil *et al.,* 2012; Saux Picart *et al.,* 2015). The simultaneous biosensor technique introduced here highlights individual differences in responses to changing environments, which can aid conservation practitioners and resource managers to better identify desired phenotypes for restoration efforts. Beyond climate-smart restoration, the approach introduced in this study could readily be applied to other applications such as assessing phenotypes more resilient to anthropogenic pollution currently impacting wild bivalve populations and to select better aquaculture broodstock (Fearman and Moltschaniwskyj, 2010; Filgueira *et al.,* 2016; Fuentes-Santos *et al.,* 2021).

## Supplementary Material

Web_Material_coaf023

## Data Availability

Raw data are available upon request to the corresponding author.
