# Supplementary material for: Determining physiological responses of mussels (Mytilus edulis) to hypoxia by combining multiple sensor techniques
Source: Conserv Physiol. 2025 Apr 11;13(1):coaf023. doi: 10.1093/conphys/coaf023 (PMC11991691; doi:10.1093/conphys/coaf023)
Supplement: Web_Material_coaf023 [file web_material_coaf023.zip › SuppMat_Petersonetal.pdf]

Supplementary Materials

Determining physiological responses of mussels (*Mytilus edulis*) to hypoxia by combining multiple sensor techniques.

Emily Adria Peterson[1,2*], Marinus Cornelis Keur[3], Michael Yeboah[2], Thomas van de Grootevheen[2], Luke Moth[2], Pauline Kamermans[3],Tinka Murk[2], Myron A. Peck[1,2], Edwin Foekema[1,2]

1. Royal Netherlands Institute for Sea Research, Department of Coastal Systems, Den Burg, Netherlands
2. Wageningen University & Research, Marine Animal Ecology, P.O. Box 338, 6700 AH Wageningen, Netherlands
3. Wageningen Marine Research, Den Helder, Netherlands

*Corresponding Author: emily.peterson@wur.nl, P.O. Box 338, 6700 AH Wageningen, Netherlands https://orcid.org/0000-0002-9691-2420

Supplementary Figures

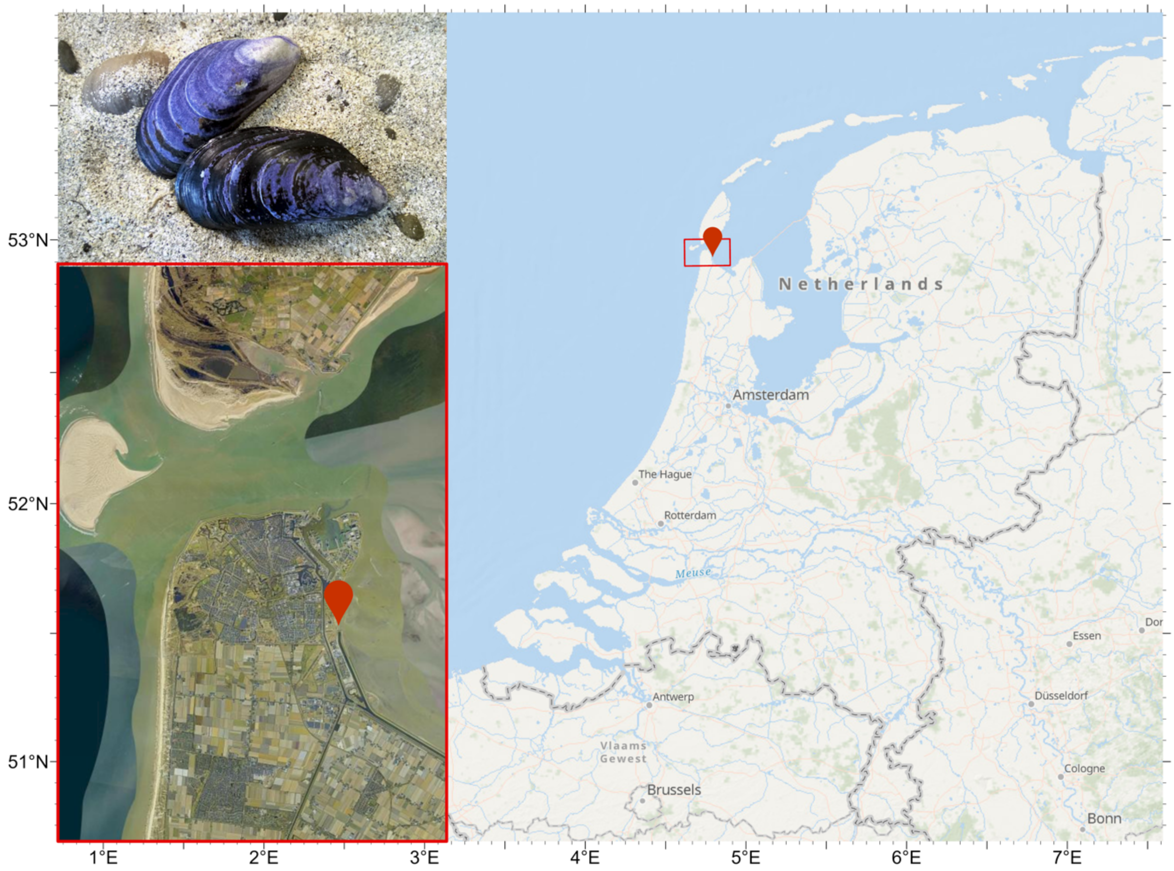

Figure S1. Study species and study area.

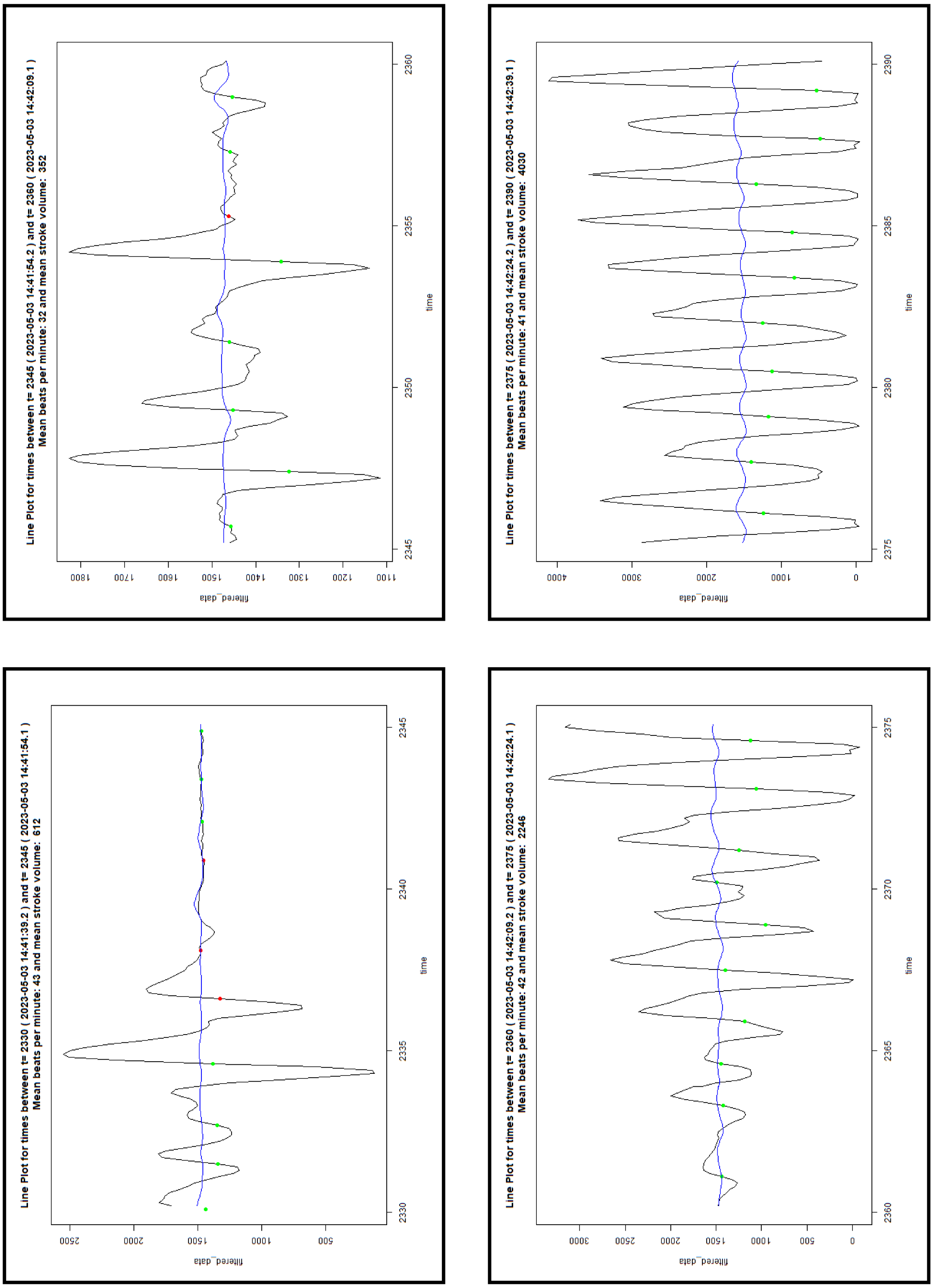

Figure S2. Zero crossing algorithm counting mechanism for beats per minute.

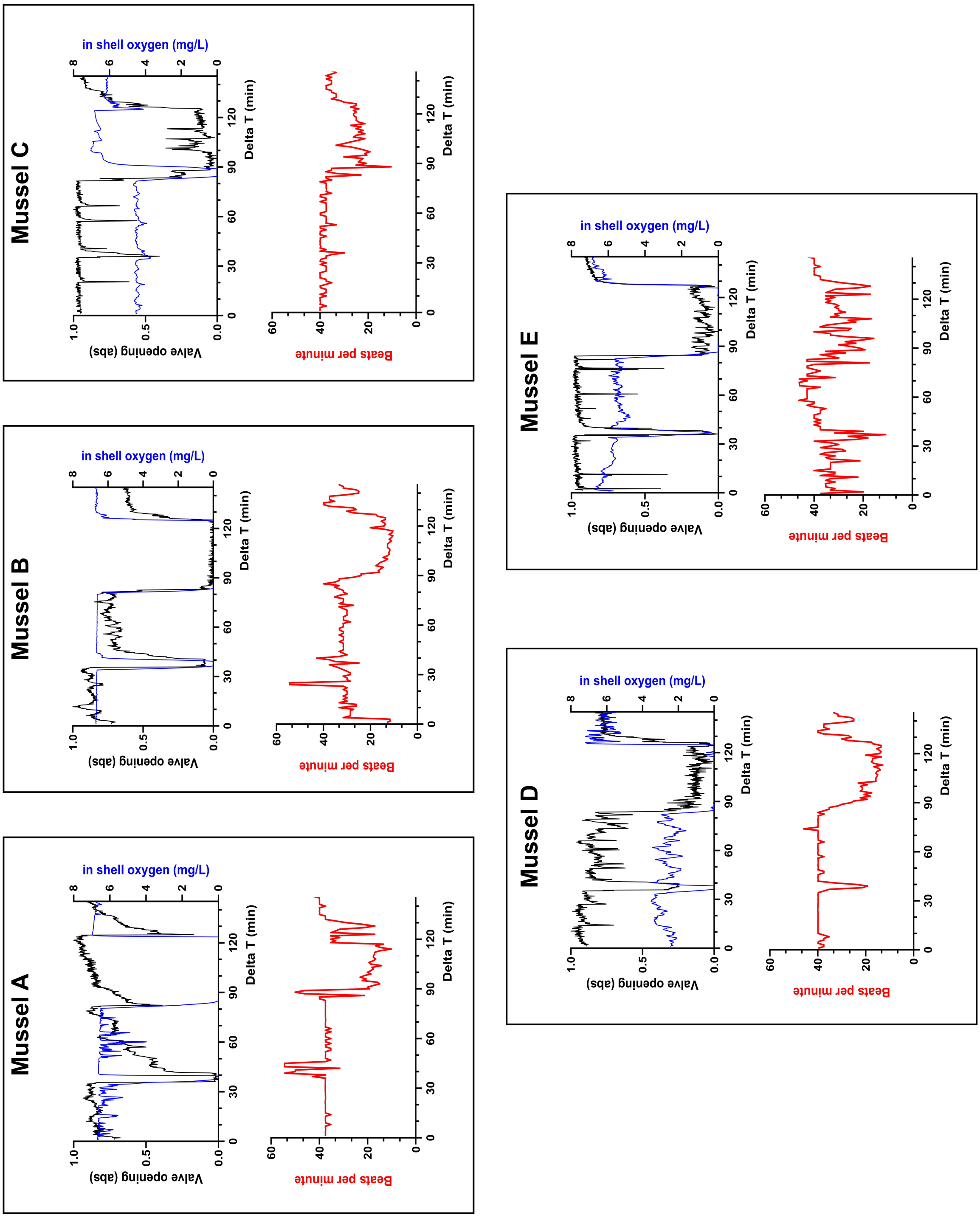

Figure S3. Mussel valve gap activity, heartbeat per minute, and in shell oxygen concentration during experiment 1a when submerged (0-75 min, 116-145 min) and dry (76-114 min).

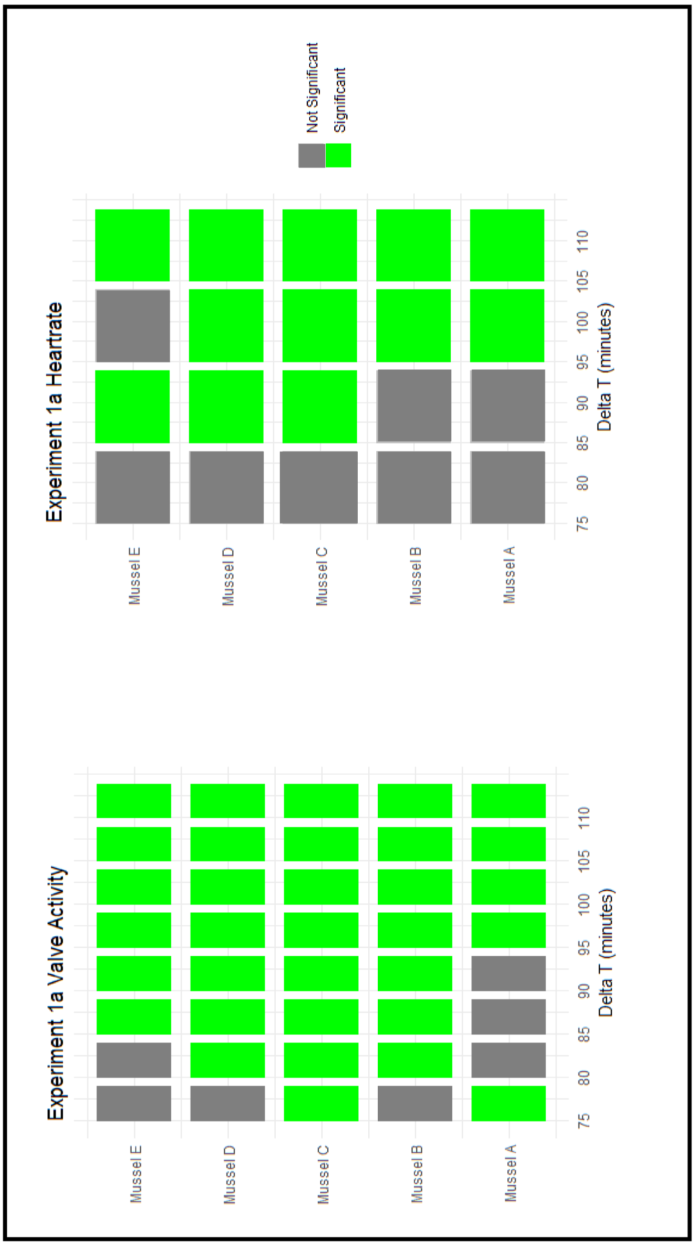

Figure S4. Statistical significance *($p< 0.05$)* of valve and heart activity during experiment 1a in comparison to the representative submerged oxygenated period (0-74 min). For full p-values, see tables S1 and S2 for valve and heartrate, respectively.

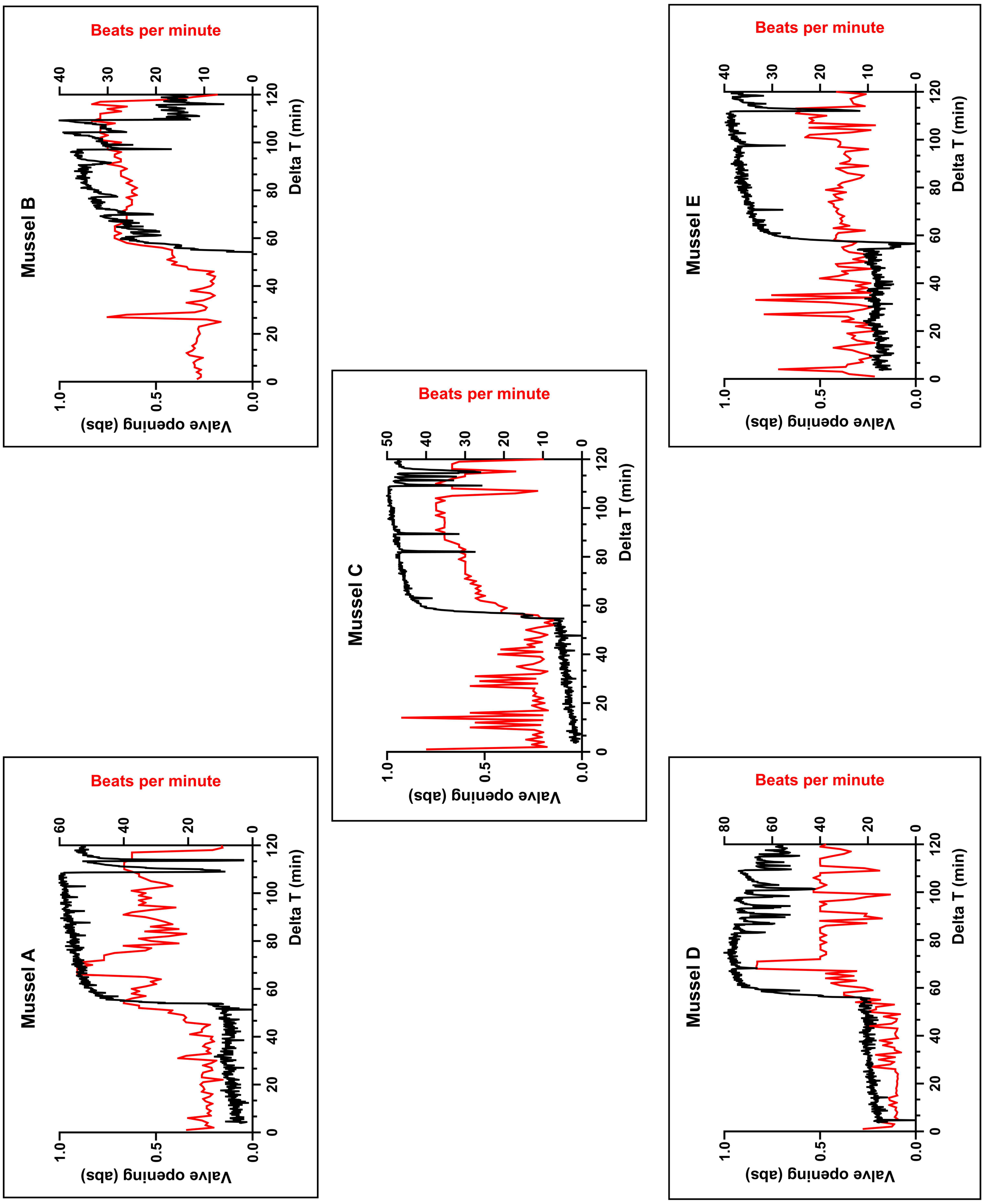

Figure S5. Mussel valve activity and heartbeat per minute during dry (0-55 min) and submerged (55-130 min) periods during experiment 1b.

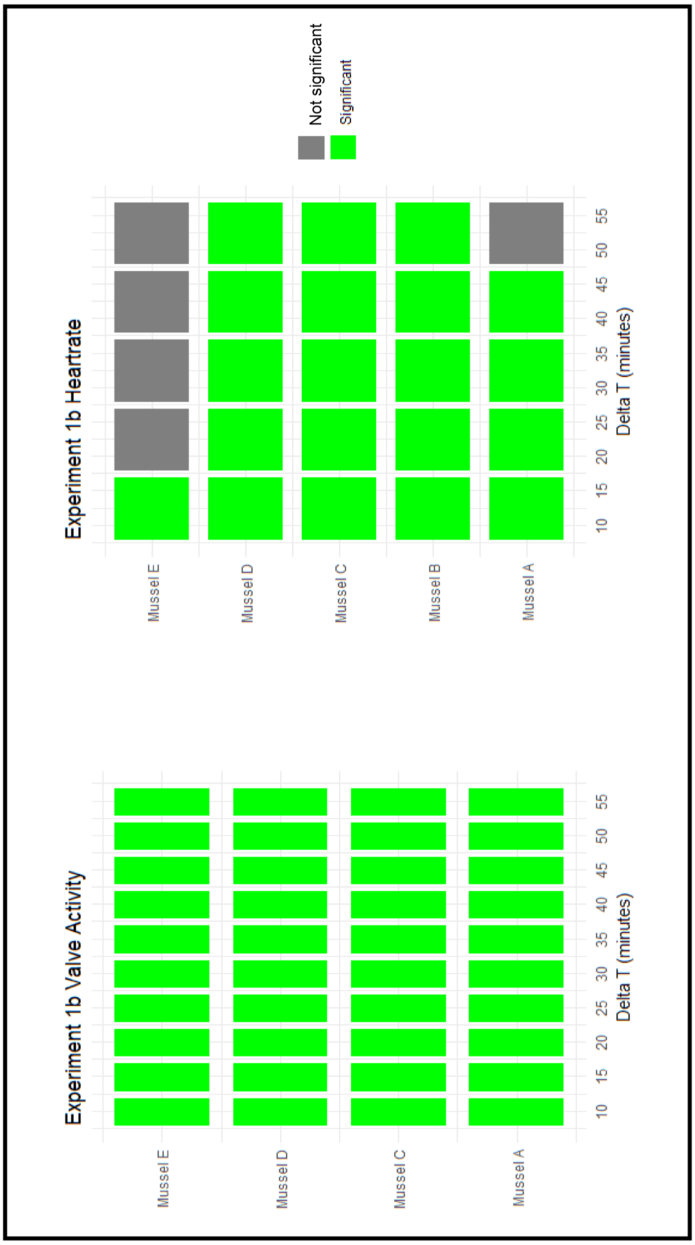

Figure S6. Statistical significance *($p< 0.05$)* of valve and heart activity during experiment 1b in comparison to the representative submerged oxygenated period (64-94 min) during experiment 1b. For full p-values, see tables S3 and S4 for valve and heartrate, respectively.

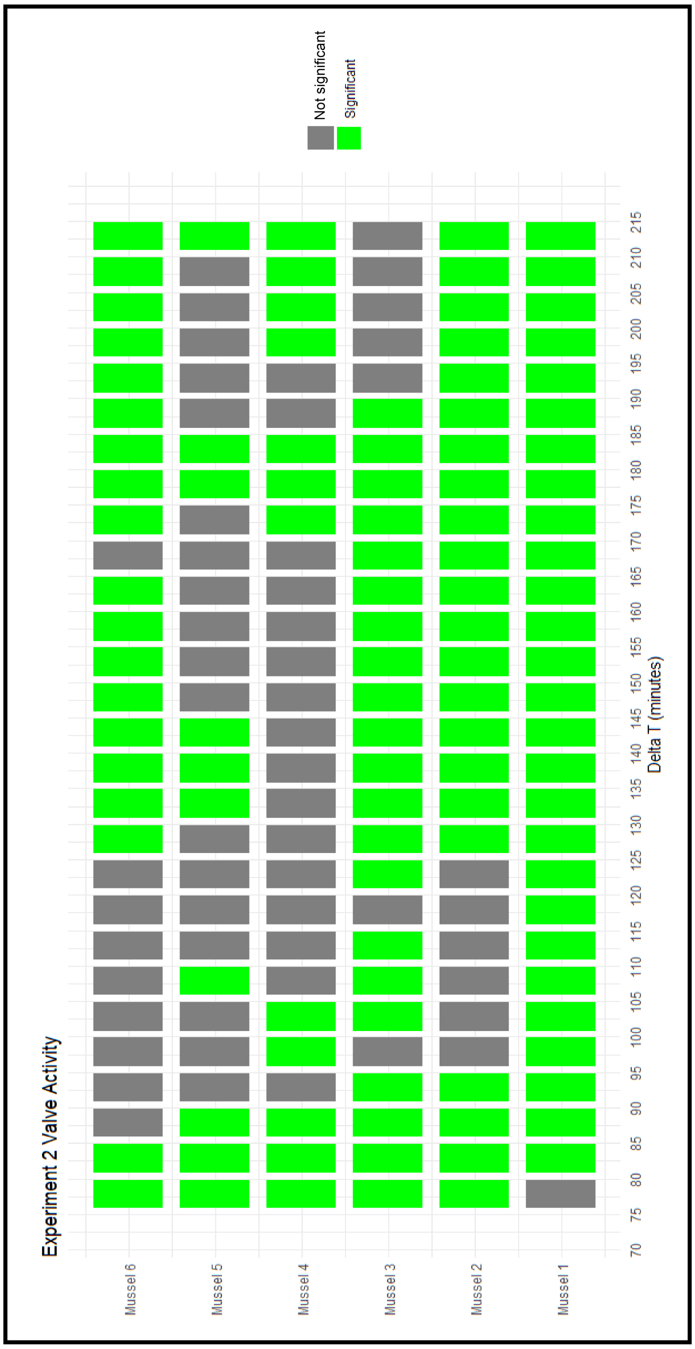

Figure S7. Statistical significance *($p < 0.05$)* of valve gape activity during experiment 2 in comparison to the representative submerged oxygenated period (0-75 min). For full p-values, see table S5.

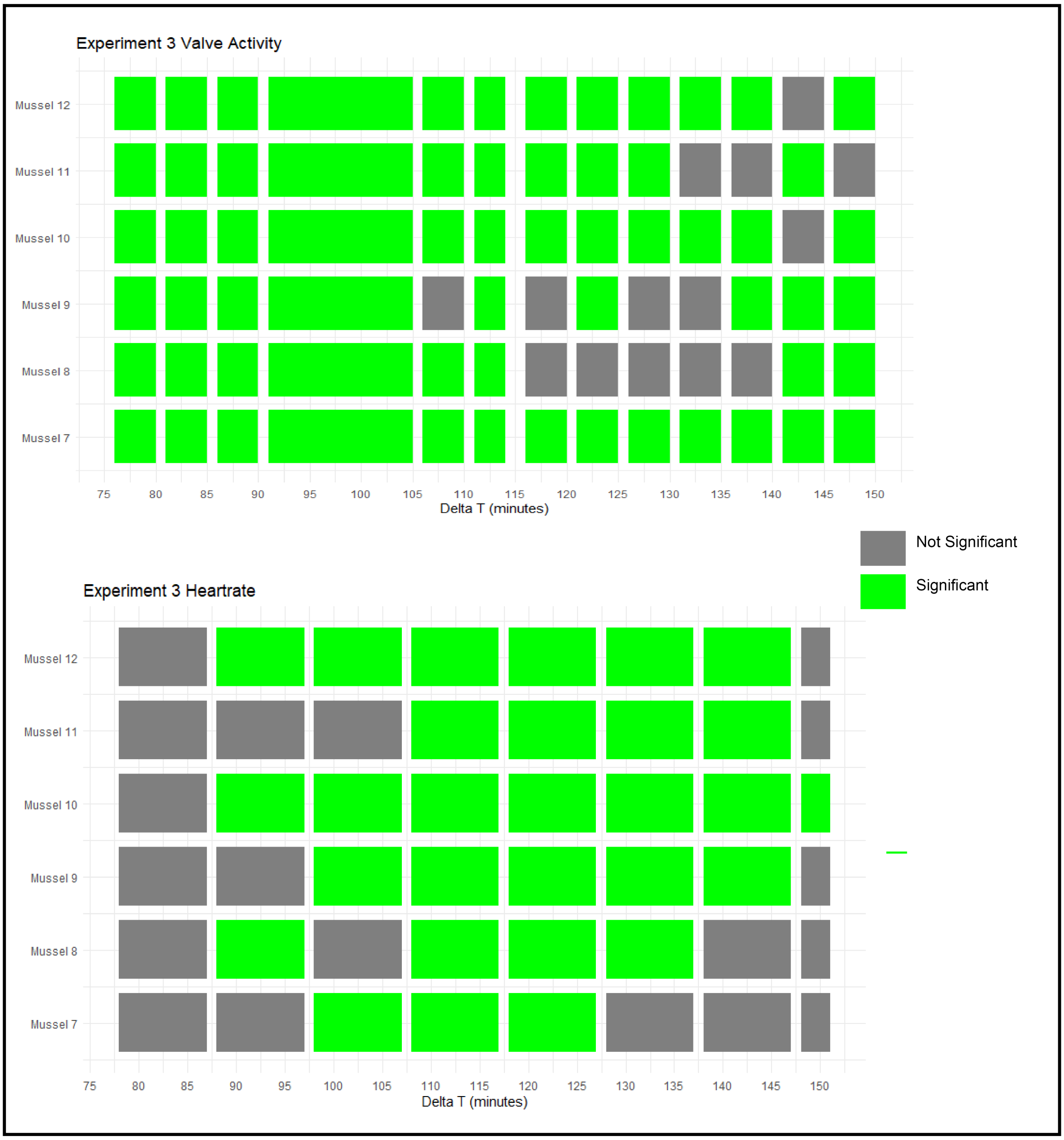

Figure S8. Statistical significance *($p < 0.05$)* of valve and heart activity during experiment 3 in comparison to the representative submerged oxygenated period (0-75 min). For full p-values, see tables S6 and S7 for valve and heartrate, respectively.

Supplementary Tables

Full p-value tables per experiment

Experiment 1a

Table S1. Adjusted p values for dry periods with significant difference in valve gap activity for all mussels in comparison to representative submerged period (0-74 min).

Delta T range (min)	Mussel A	Mussel B	Mussel C	Mussel D	Mussel E
75-79	2.76E-05	*p>0.05*	1.32E-02	*p>0.05*	*p>0.05*
80-84	*p>0.05*	2.28E-04	1.05E-03	4.36E-05	*p>0.05*
85-89	*p>0.05*	8.79E-17	2.92E-16	1.09E-14	3.71E-15
90-94	*p>0.05*	3.54E-20	5.98E-27	5.30E-19	3.20E-15
95-99	9.11E-03	2.10E-19	1.75E-23	1.01E-20	7.27E-21
100-104	1.78E-07	3.61E-21	3.77E-14	5.20E-22	5.34E-20
105-109	1.36E-12	1.67E-21	1.39E-18	1.32E-21	2.12E-15
110-114	3.13E-21	4.96E-22	1.34E-16	9.17E-25	1.81E-17

Table S2. Adjusted p values for dry periods with significant difference in heartbeats per minute activity for all mussels in comparison to representative submerged period (0-74 min).

Delta T range (min)	Mussel A	Mussel B	Mussel C	Mussel D	Mussel E
75-84	*p>0.05*	*p>0.05*	*p>0.05*	*p>0.05*	*p>0.05*
85-94	*p>0.05*	*p>0.05*	3.58E-05	8.19E-04	0.011337
95-104	3.35E-05	1.18E-04	9.49E-07	1.21E-05	*p>0.05*
105-114	6.67E-07	1.42E-06	4.76E-07	2.69E-08	0.003956

Experiment 1b

Table S3. Adjusted p values for dry periods with significant difference in valve gap activity for all mussels in comparison to representative submerged period (64-94 min).

Delta T range (min)	Mussel A	Mussel C	Mussel D	Mussel E
8-12	1.62E-33	5.06E-37	1.39E-35	1.06E-27
13-17	6.85E-27	2.71E-33	2.29E-32	1.10E-27
18-22	1.70E-22	1.91E-24	1.38E-25	9.52E-20
23-27	1.05E-17	4.70E-23	1.51E-19	1.65E-10
28-32	8.23E-10	6.81E-18	9.53E-16	1.35E-17
33-37	1.32E-12	1.79E-13	2.18E-09	2.18E-13
38-42	3.65E-18	2.02E-10	6.22E-11	2.73E-23
43-47	5.92E-18	2.85E-08	6.63E-12	1.34E-16
48-52	1.30E-09	8.67E-07	7.29E-10	5.51E-08
53-57	2.69E-02	3.19E-02	6.41E-03	1.36E-04

Table S4. Adjusted p values for dry periods with significant difference in heartbeat per minute for all mussels in comparison to representative submerged period (64-94 min).

Delta T range (min)	Mussel A	Mussel B	Mussel C	Mussel D	Mussel E
8-17	8.93E-06	1.73E-04	1.95E-03	1.11E-06	0.01862
18-27	6.65E-05	2.24E-05	1.21E-04	5.71E-08	*p>0.05*
28-37	4.25E-05	5.12E-06	2.60E-04	7.82E-05	*p>0.05*
38-47	1.52E-04	4.79E-08	6.02E-05	1.90E-06	*p>0.05*
48-57	*p>0.05*	4.36E-02	7.36E-07	1.08E-02	*p>0.05*

Experiment 2

Table S5. Adjusted p values for aqueous hypoxic periods with significant difference in valve gap activity for all mussels in comparison to representative 100% oxygen saturation period (0-75 min).

Delta T range (min)	Mussel 1	Mussel 2	Mussel 3	Mussel 4	Mussel 5	Mussel 6
76-80	*p>0.05*	3.95E-07	1.75E-11	4.43E-12	4.93E-12	1.43E-08
81-85	1.13E-02	1.74E-04	2.42E-10	1.48E-13	9.35E-08	1.53E-05
86-90	1.41E-02	2.52E-02	1.07E-08	4.02E-12	2.38E-08	*p>0.05*
91-95	1.89E-03	3.11E-04	6.88E-07	*p>0.05*	*p>0.05*	*p>0.05*
96-100	6.92E-06	*p>0.05*	*p>0.05*	9.73E-05	*p>0.05*	*p>0.05*
101-105	6.80E-08	*p>0.05*	4.37E-07	4.66E-06	*p>0.05*	*p>0.05*
106-110	5.71E-10	*p>0.05*	4.41E-06	*p>0.05*	6.68E-15	*p>0.05*
111-115	6.08E-18	*p>0.05*	8.36E-04	*p>0.05*	*p>0.05*	*p>0.05*
116-120	4.31E-17	*p>0.05*	*p>0.05*	*p>0.05*	*p>0.05*	*p>0.05*
121-125	5.17E-24	*p>0.05*	2.43E-04	*p>0.05*	*p>0.05*	*p>0.05*
126-130	4.80E-23	3.92E-03	3.75E-03	*p>0.05*	*p>0.05*	1.89E-16
131-135	3.73E-37	2.14E-17	1.50E-04	*p>0.05*	2.55E-09	7.06E-10
136-140	5.18E-37	1.89E-17	1.57E-07	*p>0.05*	2.47E-04	6.45E-11
141-145	1.61E-35	1.96E-16	2.64E-11	*p>0.05*	3.77E-02	5.14E-13
146-150	1.11E-35	3.83E-08	8.79E-15	*p>0.05*	*p>0.05*	3.81E-08
151-155	2.29E-34	2.62E-07	4.75E-15	*p>0.05*	*p>0.05*	3.13E-04
156-160	5.24E-31	2.19E-11	1.17E-16	*p>0.05*	*p>0.05*	5.96E-04
161-165	4.89E-30	3.39E-16	1.29E-15	*p>0.05*	*p>0.05*	3.16E-04
166-170	1.30E-25	1.54E-21	3.28E-13	*p>0.05*	*p>0.05*	*p>0.05*
171-175	4.56E-26	1.43E-23	1.70E-12	7.50E-04	*p>0.05*	4.64E-02
176-180	4.31E-36	8.49E-23	3.94E-12	4.08E-08	9.18E-08	5.52E-04
181-185	2.35E-18	6.78E-21	2.24E-11	7.40E-05	1.37E-02	1.31E-02
186-190	1.79E-08	1.61E-08	9.53E-04	*p>0.05*	*p>0.05*	1.82E-18
191-195	2.12E-06	1.63E-07	*p>0.05*	*p>0.05*	*p>0.05*	9.70E-20
196-200	1.36E-05	3.90E-08	*p>0.05*	5.42E-05	*p>0.05*	1.68E-11
201-205	1.76E-10	2.47E-08	*p>0.05*	3.04E-10	*p>0.05*	3.47E-14
206-210	3.78E-12	3.09E-15	*p>0.05*	1.47E-09	*p>0.05*	1.51E-18
211-215	5.63E-06	1.10E-09	*p>0.05*	3.90E-05	9.35E-08	9.41E-11

Experiment 3

Table S6. Adjusted p values for aqueous hypoxic periods with significant difference in valve gap activity for all mussels in comparison to representative 100% oxygen saturation period (0-75 min).

Delta T range (min)	Mussel 7	Mussel 8	Mussel 9	Mussel 10	Mussel 11	Mussel 12
76-80	4.39E-06	6.45E-05	1.68E-04	1.04E-04	5.15E-24	1.04E-04
81-85	7.90E-13	6.55E-11	2.08E-13	9.57E-25	4.52E-16	9.57E-25
86-90	4.86E-13	1.10E-06	6.91E-19	9.54E-28	7.66E-24	9.54E-28
91-96	2.13E-15	1.63E-08	7.02E-10	1.15E-22	2.06E-23	1.15E-22
96-101	1.59E-14	2.72E-09	1.52E-15	6.53E-21	1.76E-08	6.53E-21
101-105	3.34E-28	2.15E-04	6.05E-07	3.28E-22	3.30E-09	3.28E-22
106-110	1.39E-23	2.23E-05	*p>0.05*	1.47E-19	8.35E-13	1.47E-19
111-115	4.53E-03	1.12E-05	4.42E-06	2.85E-15	3.29E-02	2.85E-15
116-120	4.39E-14	*p>0.05*	*p>0.05*	6.05E-11	2.83E-10	6.05E-11
121-125	1.68E-23	*p>0.05*	1.81E-02	3.28E-10	8.49E-06	3.28E-10
126-130	1.26E-30	*p>0.05*	*p>0.05*	9.66E-04	3.13E-16	9.66E-04
131-135	2.79E-06	*p>0.05*	*p>0.05*	1.04E-02	*p>0.05*	1.04E-02
136-140	1.39E-03	*p>0.05*	7.69E-08	1.64E-02	*p>0.05*	1.64E-02
141-145	8.48E-06	2.00E-04	5.69E-12	*p>0.05*	7.90E-04	*p>0.05*
146-150	1.71E-03	1.56E-05	3.14E-11	6.94E-03	*p>0.05*	6.94E-03

Table S7. Adjusted p values for aqueous hypoxic periods with significant difference in heartbeats per minute for all mussels in comparison to representative 100% oxygen saturation period (0-75 min).

Delta T range (min)	Mussel 7	Mussel 8	Mussel 9	Mussel 10	Mussel 11	Mussel 12
78-87	*p>0.05*	*p>0.05*	*p>0.05*	*p>0.05*	*p>0.05*	*p>0.05*
88-97	*p>0.05*	1.69E-02	*p>0.05*	2.29E-02	*p>0.05*	2.34E-02
98-107	2.82E-04	*p>0.05*	2.36E-04	1.80E-03	*p>0.05*	4.92E-04
108-117	1.37E-04	2.45E-05	7.28E-06	5.75E-04	4.84E-03	1.22E-03
118-127	3.35E-03	3.12E-05	5.16E-08	4.49E-06	3.22E-05	7.39E-07
128-137	*p>0.05*	1.44E-02	1.04E-08	4.06E-09	2.21E-06	1.39E-05
138-147	*p>0.05*	*p>0.05*	4.43E-04	8.26E-09	2.03E-07	6.58E-07
148-151	*p>0.05*	*p>0.05*	*p>0.05*	1.85E-02	*p>0.05*	*p>0.05*
